# Directional asymmetry and direction‐giving factors: Lessons from flowers with complex symmetry

**DOI:** 10.1111/ede.12402

**Published:** 2022-06-16

**Authors:** Sanja Budečević, Sanja Manitašević Jovanović, Ana Vuleta, Branka Tucić, Christian Peter Klingenberg

**Affiliations:** ^1^ Department of Evolutionary Biology, Institute for Biological Research “Siniša Stanković”—National Institute of the Republic of Serbia University of Belgrade Belgrade Serbia; ^2^ School of Biological Sciences University of Manchester Manchester UK

**Keywords:** adaxial–abaxial polarity, directional asymmetry, direction‐giving factors, fluctuating asymmetry, geometric morphometrics, *Iris pumila*, target phenotype

## Abstract

Directional asymmetry is a systematic difference between the left and right sides for structures with bilateral symmetry or a systematic differentiation among repeated parts for complex symmetry. This study explores factors that produce directional asymmetry in the flower of *Iris pumila*, a structure with complex symmetry that makes it possible to investigate multiple such factors simultaneously. The shapes and sizes of three types of floral organs, the falls, standards, and style branches, were quantified using the methods of geometric morphometrics. For each flower, this study recorded the compass orientations of floral organs as well as their anatomical orientations relative to the two spathes subtending each flower. To characterize directional asymmetry at the whole‐flower level, differences in the average sizes and shapes according to compass orientation and relative orientation were computed, and the left–right asymmetry was also evaluated for each individual organ. No size or shape differences within flowers were found in relation to anatomical position; this may relate to the terminal position of flowers in *Iris pumila*, suggesting that there may be no adaxial–abaxial polarity, which is very prominent in many other taxa. There was clear directional asymmetry of shape in relation to compass orientation, presumably driven by a consistent environmental gradient such as solar irradiance. There was also clear directional asymmetry between left and right halves of every floral organ, most likely related to the arrangement of organs in the bud. These findings indicate that different factors are acting to produce directional asymmetry at different levels. In conventional analyses not recording flower orientations, these effects would be impossible to disentangle from each other and would probably be included as part of fluctuating asymmetry.

## Research Highlights

Structures with complex symmetry can have several types of directional asymmetry, each arising through different mechanisms and in response to different direction‐giving factors. This study uses flowers or *Iris pumila* to demonstrate this concept empirically.

## Graphical abstract

The flowers od *Iris pumila* have a complex symmetry. This makes it possible to study three distinct types of directional asymmetry, originating from different direction‐giving factors: left–right asymmetry within individual parts and asymmetry over the whole flower in relation to either anatomical orientation or compass orientation.

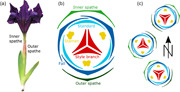

## 1 INTRODUCTION

Directional asymmetry is defined as a consistent asymmetry, so that traits on the left and right sides within individuals differ in a systematic way, and accordingly the average left–right asymmetry differs from zero (Palmer & Strobeck, 1986; Van Valen, 1962). Alternatively, it can be defined using the concept of the target phenotype, the phenotype that is expected for a structure of interest for a specific genotype and environment (Nijhout & Davidowitz, 2003). Using this concept, directional asymmetry can be defined as a difference in the target phenotypes between the left and right sides (Klingenberg, 2019). Directional asymmetry has long been known but generally has received less attention than fluctuating asymmetry, the small random deviations from symmetry (Møller & Swaddle, 1997; Palmer & Strobeck, 1986; Polak, 2003; Van Dongen, 2006). Most animals have obvious directional asymmetries of their internal organs, even though they may externally appear to be symmetric (Levin, 2005; Wood, 1997). Nevertheless, directional asymmetry is also widespread for external structures even in organisms that superficially appear to be symmetric. Especially since geometric morphometric methods have been used to quantify the asymmetry of shape, directional asymmetry has been found in the vast majority of studies conducted in animals and less consistently in plants (e.g., Auffray et al., 1996; Chitwood et al., 2012; Klingenberg, 2015; Klingenberg et al., 1998; Savriama et al., 2012; Tucić et al., 2018). It is widely held that directional asymmetry is developmentally controlled and has a genetic basis (e.g., Leamy, 1984; Palmer & Strobeck, 1992; Van Valen, 1962), but its developmental origins and evolutionary significance are not well understood.

For structures with bilateral symmetry, the left and right sides are unambiguous, and therefore few questions arise concerning the nature and interpretation of directional asymmetry. By contrast, for structures with complex symmetry, such as radial symmetry, it is less clear how directional asymmetry should be considered. A structure with complex symmetry consists of multiple parts that are repeated in different relative orientations and positions, for instance, the petals of a flower, and this arrangement of parts is characteristic of each type of symmetry (Klingenberg, 2015; Savriama, 2018; Savriama & Klingenberg, 2011). With complex symmetry, directional asymmetry means that the repeated parts that form the overall structure differ systematically from each other, which, in turn, implies that different parts have different target phenotypes. It is not clear, however, what factors may cause them to differ. To induce consistent differences among target phenotypes, such factors must relate consistently to the relevant directions in the structure. There might even be more than one kind of directional asymmetry, corresponding to different factors that can cause differences in target phenotypes among the repeated parts of a structure. We call such factors “direction‐giving factors.”

Flowers have a wide range of different types of symmetry, including zygomorphic flowers that have bilateral symmetry as well as complex symmetries such as disymmetry, rotational and radial symmetry (Citerne et al., 2010; Endress, 1999, 2001). Depending on the type of symmetry, multiple components of asymmetry may exist (Klingenberg, 2015; Savriama, 2018; Savriama et al., 2012; Savriama & Klingenberg, 2011), including directional asymmetries corresponding to different direction‐giving factors. The most prominent and best known direction‐giving factor of flowers is the adaxial–abaxial (or dorsal–ventral) polarity, which relates to the anatomical structure and development of flowers and for which the involvement of *CYCLOIDEA* and associated genes has been studied extensively (Kim et al., 2008; Luo et al., 1996; Nakagawa et al., 2020; Preston & Hileman, 2009; Spencer & Kim, 2018). Directional asymmetry in the adaxial–abaxial direction is overwhelming in zygomorphic flowers (to the extent that it is often impossible to quantify), but has also been found in a more subtle form in disymmetric flowers (Savriama et al., 2012). The organization of the flower and the developmental processes by which its parts originate can themselves produce inherent directional asymmetry in those parts, and therefore may act as direction‐giving factors. For example, rotationally symmetric flowers often have petals that are each noticeably asymmetric, resulting in a “pinwheel symmetry” of the whole flower (Endress, 1999, 2001). In this case, the direction of rotation and convolute arrangement of parts in the flower bud act as the direction‐giving factor; if the direction of rotation is constant in a population, this produces directional asymmetry of the petals. Finally, phenotypic plasticity can produce asymmetries in response to environmental heterogeneity. If an environmental gradient exists at a sufficiently large scale, so that it affects all flowers in a population in the same directed manner (e.g., solar irradiance), consistent asymmetries may result (Tucić et al., 2018). A consistent asymmetry of this kind also is directional asymmetry, and the environmental gradient is the direction‐giving factor. Identifying these different kinds of directional asymmetry can be challenging and requires recording information that empirical studies usually do not collect. For instance, characterizing asymmetry from plasticity in response to an environmental gradient requires information about the orientation of each flower in relation to the gradient. Without this information, any such asymmetry would be captured as part of fluctuating, not directional asymmetry.

**Figure 1:**
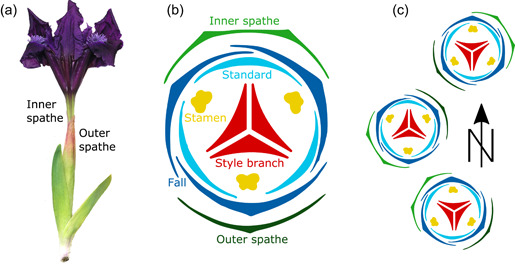
Experimental design of this study. (a) A flower of *Iris pumila*, showing the two spathes subtending it, along with two leaves. The outer spathe almost completely envelops the inner one, of which only the uppermost part is visible to the left. (b) Floral diagram, showing the arrangement of floral parts in relation to the spathes (modified after Eichler, 1875; Guo & Wilson, 2018; Remizowa et al., 2013). Note that each of the floral organs is bilaterally symmetric (with an axis of symmetry going through its middle and the center of the flower). (c) The positions of floral parts and outer spathes were recorded, so that both the compass orientation of every floral organ and its orientation relative to the outer spathe was known. [Color figure can be viewed at wileyonlinelibrary.com]

The idea of direction‐giving factors and the distinction between corresponding types of directional asymmetry are introduced for the first time in this paper. Previous investigators have not recorded the required information and thus unknowingly subsumed some or all of the effects in the estimate of fluctuating asymmetry. To characterize separately the types of directional asymmetry due to the three different direction‐giving factors mentioned above, this study replicates an earlier investigation in flower organs of *Iris pumila* (Tucić et al., 2018) and extends its design by including anatomical orientation as an additional factor. This species has single flowers in a terminal position on short shoots, so that the adaxial–abaxial polarity of flowers is not obvious. Nevertheless, recording the orientation of the two spathes subtending each flower provides unambiguous information about the anatomical polarity of flowers (Figure 1a,b). The insight that at least three different types of directional asymmetry due to different direction‐giving factors can be distinguished leads to some general considerations about directional asymmetry and the differentiation of parts that have far‐reaching implications for understanding complex organismal structures.

## 2 MATERIALS AND METHODS

### 2.1 Study species, experimental design, and collection of samples

*Iris pumila* L. is a perennial plant persisting as rhizomes (modified stems positioned horizontally, partly above the ground) and forming clones of variable sizes, depending on the age of the clone (Tucić et al., 1989). Each flowering ramet produces a very short stem with a single terminal flower. Flowers are actinomorphic and consist of four whorls, each made up of three parts: the petaloid sepals, which are called “falls,” the petals, called “standards,” the stamens, and the gynoecium with three petaloid style branches (Guo & Wilson, 2018; Mathew, 1989; Pande & Singh, 1981; Webb & Chater, 1980). The basal portions of the falls, standards, and anthers are fused into a lengthened hypanthial tube (Webb & Chater, 1980). The flower is subtended by two spathes that envelop the flower bud during early development. In relation to the floral ground plan in *Iris* (and more generally in Iridaceae; Eichler, 1875; Remizowa et al., 2013), the outer (and lower) spathe can be interpreted as the bract subtending the entire flower and the inner (and upper) spathe as the floral prophyll. Accordingly, the perianth is expected to be oriented so that one of the falls is positioned directly above the outer spathe and one of the standards directly above the inner spathe (Figure 1b).

The plants used in this study originated from hand‐pollinated crosses of plants from a natural population performed in 1996 and were subsequently grown in clay pots in a common garden located on the grounds of the Institute for Biological Research “Siniša Stanković” in Belgrade (for further details, see Manitašević Jovanović et al., 2011; Tucić et al., 2013). Each pot contained ramets of one genet established from a single seedling from one of the initial crosses. Pots were haphazardly placed and not moved during the period of flower development.

Flowers were harvested in the period from March 29 to April 5, 2016, following the protocol described in Tucić et al. (2018). The compass orientation for each harvested flower was recorded to the nearest 60°, according to whether one of the falls or one of the standards was facing approximately south; compass orientations of individual flower parts were calculated accordingly, with 0° designating a southerly direction (for additional detail, see Tucić et al., 2018). As far as possible, two flowers from each clone were harvested, one with one of the falls approximately facing south, the other with a standard approximately facing south (i.e., with a difference in orientation by ca. 60°). In addition, for each flower in this experiment, the orientation of the outer (lower) of the two spathes was also recorded to the nearest 60° to provide information on the anatomical directions for each flower. Flowers were stored in 70% ethanol immediately after being harvested.

Flowers were dissected by cutting at the distal end of the floral tube to separate the floral organs. The falls, standards, and style branches of each flower were spread out on a glass plate coated with 50% glycerol, keeping track of the original orientation of each part, and digital images were obtained with a flatbed scanner (for further details, see Tucić et al., 2018).

### 2.2 Landmark data and morphometric analyses

**Figure 2:**
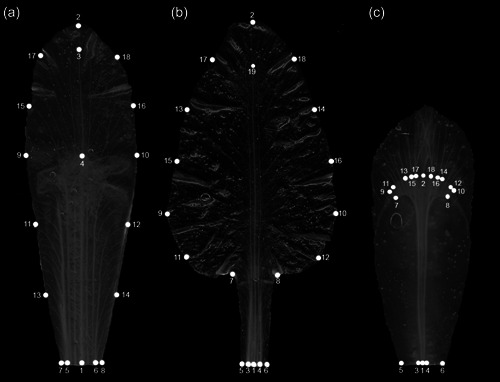
The three floral organs included in this study and the landmarks digitized for each of them. (a) Standard. (b) Fall. (c) Style branch. Reproduced with permission from Tucić et al. (2018).

The landmarks used in this study (Figure 2) are the same as those used in Tucić et al. (2018) and were digitized from the images by one person, using a custom‐written plug‐in and the ImageJ software (Schneider et al., 2012). In brief, the shape of the falls was characterized by 18 landmarks arranged at the base, on the central nerve, and along the margins (Figure 2a). For the standards, there were 19 landmarks at the base and surrounding the broadened blade, and also at the first branching point of the central nerve near the apex (Figure 2b). For the style branches, 18 landmarks were digitized at the base and on the stigma, but it was not possible to locate landmarks consistently on the terminal lobes because of the extreme variation in this region (Figure 2c). This study includes landmark data for the three sets of floral organs of 462 flowers from 314 plants.

Because we dissected the flowers to separate the individual floral organs (Figure 2), we are considering the symmetry at the level of the whole flower as matching symmetry (Klingenberg, 2015; Savriama, 2018; Savriama & Klingenberg, 2011). Accordingly, directional asymmetry of shape at the whole‐flower level can be tested and characterized by examining differences in the mean shapes of flower parts according to their different positions within the respective whorl. In addition, each of the three structures included in this study, when considered as a separate part, is bilaterally symmetric (Figure 2). Therefore, each floral organ can be analyzed with the framework for bilateral object symmetry (Klingenberg et al., 2002; Savriama & Klingenberg, 2011). This yields separate symmetric and asymmetric components of shape variation that can be compared at the whole‐flower level and may each provide different insights, and it also provides the opportunity to investigate a further level of directional asymmetry within individual flower organs (Tucić et al., 2018).

The study design makes it possible to investigate three different types of directional asymmetry and to make inferences about the respective direction‐giving factors: (i) the anatomical polarity of the flowers, which concerns the orientation of flower parts relative to the outer spathe, (ii) the directional asymmetry in response to consistent environmental gradients such as solar irradiance, which is associated with the compass orientation of flower parts, and (iii) the directional asymmetry of each individual flower organ, from the analyses of bilateral object symmetry within each landmark configuration. The first two of these types are analyzed by comparisons at the whole‐flower level, grouping flower organs by their orientation relative to the outer spathe for type (i) or by their compass orientation for type (ii), whereas type (iii) uses the standard analysis of bilateral object symmetry simultaneously within each of the floral organs of a whorl (i.e., with asymmetry computed from the difference between the original landmark configurations and a reflected and relabeled copy of each of them; Klingenberg et al., 2002). The relative orientation of a floral organ was computed by subtracting the orientation of the spathe for that flower from the compass orientation of the floral organ of interest and, if the result was negative, adding 360° (i.e., the angle between the spathe and the organ in question, measured in a clockwise direction).

To test whether flower organs with different compass orientations or relative orientations differed in their centroid size, we ran separate one‐way analyses of variance (ANOVAs) for the falls, standards, and style branches, with the respective orientations as the grouping criterion. To evaluate the effects on the shape of flower organs, we used canonical variate analysis (CVA) with groups defined either by compass orientation or relative orientation. Canonical variates (CVs) maximize the difference among groups relative to the variation within groups. As a global test of the differences, a permutation test against the null hypothesis of no differences among groups was run, using Goodall's *F* and Pillai's trace as test statistics (10,000 permutation iterations for each test). The CVAs were run separately for the symmetric and asymmetric components of shape variation for each floral organ. Differences among the average shapes for different orientations were visualized by outline drawings warped using the thin‐plate spline, amplified sufficiently to make the shape changes easily visible (Klingenberg, 2013). Analyses were run using MorphoJ (Klingenberg, 2011) and R software (R Core Team, 2021).

## 3 RESULTS

The compass orientations of floral organs were more or less evenly distributed, with 211 flowers with falls and style branches at 0°, 120°, and 240° (and standards at 60°, 180°, and 300°) and 251 flowers with falls and style branches at 60°, 180°, and 300° (and standards at 0°, 120°, and 240°). Similarly, the compass orientations of the spathes were spread over all directions (0°: 95 flowers; 60°: 73 flowers; 120°: 77 flowers; 180°: 74 flowers; 240°: 68 flowers; 300°: 75 flowers), and a Chi‐square test indicated no significant deviation from a uniform distribution (*χ* ^2^ = 3.46, *df* = 5, *p* = .34). By contrast, the orientations of flowers relative to the spathe were more concentrated: 315 of the flowers had a fall and style branch directly above the outer spathe (relative orientations of 0°, 120°, and 240° for the falls and style branches, and of 60°, 180°, and 300° for the standards), whereas only 147 flowers had an orientation relative to the spathe that differed by 60° from this arrangement (relative orientations of 60°, 180°, and 300° for the falls and style branches, and of 0°, 120°, and 240° for the standards). Therefore, just over two‐thirds of flowers were in the orientation expected from the floral ground plan (Figure 1b), but slightly less than a third were rotated by 60°.

The effects of compass orientation and relative orientation on the average centroid size of floral organs were subtle. For compass orientation, all ANOVAs produced nominally significant results (falls: *F*_5, 1372_ = 3.12, *p* = .0082; standards: *F*_5, 1372_ = 2.31, *p* = .042; style branches: *F*_5, 1374_ = 2.60, *p* = .024). Nevertheless, average differences were small (mostly about 2% of the means or less, Table 1) and suggested that floral organs tended to be slightly bigger for flowers with a fall and style branch oriented in a southerly direction (0°) than in flowers with a standard in that orientation (Table 1). For relative orientation, there were some slight but statistically nonsignificant size differences in the falls and standards between flowers with one of the falls directly above the outer spathe and flowers rotated by 60° from this arrangement, but none for the style branches (falls: *F*_5, 1372_ = 1.27, *p* = .27; standards: *F*_5, 1372_ = 2.04, *p* = .07; style branches: *F*_5, 1374_ = 0.054, *p* = .998).

**Table 1:** Size of floral organs in response to compass orientation and relative orientation

	Compass orientation			Relative orientation		
Orientation	*N*	Mean	SE	*N*	Mean	standard error (SE)
*Fall*						
0°	210	346.0	3.0	314	347.6	2.3
60°	251	353.2	2.6	146	352.7	3.7
120°	211	344.8	2.9	312	347.8	2.3
180°	248	355.1	2.6	146	353.4	3.6
240°	210	343.4	2.9	313	346.9	2.3
300°	248	351.4	2.6	147	354.2	3.6
*Standard*						
0°	247	358.8	2.9	147	359.8	3.9
60°	211	349.4	3.0	313	350.9	2.5
120°	249	358.2	3.0	146	359.2	3.9
180°	210	350.2	3.1	314	351.5	2.5
240°	251	355.0	2.9	146	359.3	4.0
300°	210	348.7	3.1	312	350.8	2.5
*Style branch*						
0°	211	214.9	1.7	314	217.2	1.3
60°	250	219.8	1.5	147	217.3	2.1
120°	211	215.4	1.7	312	217.5	1.3
180°	250	220.5	1.4	147	217.5	2.0
240°	211	215.0	1.7	313	217.5	1.3
300°	247	218.3	1.5	147	218.4	2.0

*Note*: Tabled values are the sample size (*N*), the mean centroid size in millimeters, and its SE.

**Figure 3:**
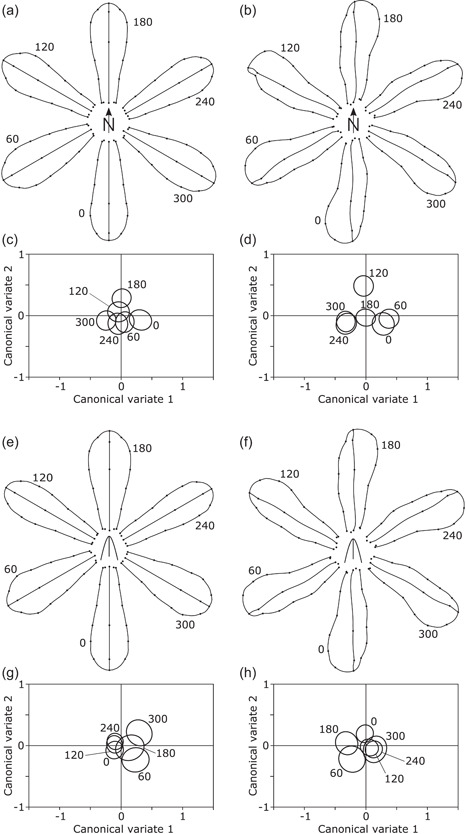
Effects of compass orientation (a–d) and orientation relative to the outer spathe (e–h) on the shape of the falls. The panels to the left (a, c, e, g) show results from analyses of the symmetric component of shape variation, whereas those to the right (b, d, f, h) display results from analyses of the asymmetric component. The diagrams of shape changes (a, b, e, f) show the differences among mean shapes for the different orientations exaggerated 15‐fold to make them more easily visible. Plots of canonical variate scores (c, d, g, h) show the 95% confidence ellipses for the means for the groups defined by compass or relative orientation.

For the falls, the averages for the symmetric component of shape differed only subtly among compass orientations. Shape changes concerned the relative lengthening or widening of the entire falls and how far the curvature of the lateral edges extended from the tip toward the base (Figure 3a). The respective CVA produced considerable overlap among the confidence regions for the average CV scores of the different compass orientations (Figure 3c). Consistent with this, permutation tests yielded somewhat ambiguous results differing according to the test statistic (Goodall's *F* = 0.81, *p* = .751; Pillai's trace = 0.078, *p* = .022). For the asymmetric component of shape variation in the falls, differentiation among compass orientations was clearer, with less overlap of confidence ellipses for the CV scores (Figure 3d) and clearly significant results in the permutation test (Goodall's *F* = 4.73, *p* < .0001; Pillai's trace = 0.152, *p* < .0001). The shape changes (Figure 3b) consisted of a conspicuous and consistent asymmetry of each fall, generating a “pinwheel symmetry” overall, combined with some shape changes specific for each specific compass orientation.

For the relative orientations, differences in the symmetric component of the shape of the falls were also very subtle, mainly involving a slight tendency for the falls at the relative positions of 0°, 120°, and 240° to be slightly narrower than those at 60°, 180°, and 300° (Figure 3e). This was also reflected in the CVA, with the former three positions tending to have slightly lower CV1 scores than the latter three (Figure 3g). These tendencies need to be interpreted cautiously, however, because the permutation test for these comparisons produced clearly nonsignificant results (Goodall's *F* = 0.940, *p* = .541; Pillai's trace = 0.048, *p* = .897). For the asymmetric component of shape, the systematic asymmetry of each fall was again evident, but there were no clear differences associated with specific orientations (Figure 3f). That relative orientation had no clear effect on the asymmetry component in the falls also was apparent from the extensive overlap of confidence regions for the CVs (Figure 3h) and from nonsignificant results in the permutation test (Goodall's *F* = 0.603, *p* = .91; Pillai's trace = 0.0619, *p* = .312).

**Figure 4:**
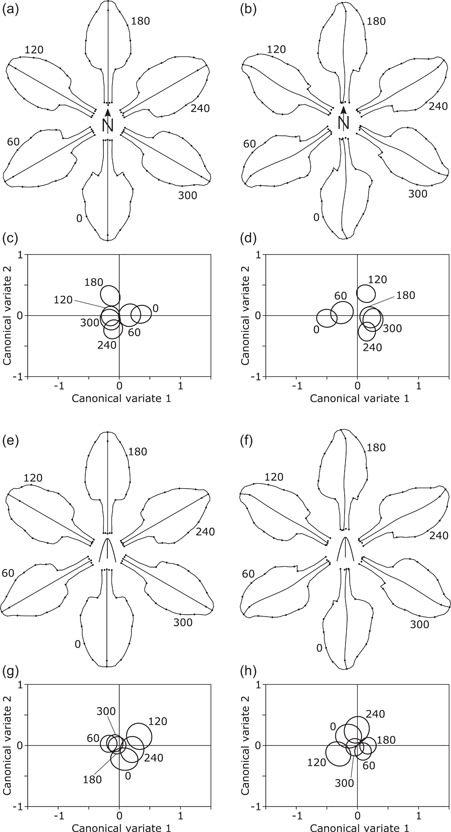
Effects of compass orientation (a–d) and relative orientation (e–h) on the shape of the standards. The panels to the left (a, c, e, g) show results from analyses of the symmetric component of shape variation, whereas those to the right (b, d, f, h) display results from analyses of the asymmetric component. The diagrams of shape changes (a, b, e, f) show the differences among mean shapes for the different orientations exaggerated 15‐fold to make them more easily visible. Plots of canonical variate scores (c, d, g, h) show the 95% confidence ellipses for the means for the groups defined by compass or relative orientation.

For the standards, there were some subtle but noticeable differences in the means of the symmetric component of shape among compass orientations, which particularly affected the basal part of the blade of the standards (Figure 4a). This corresponds to a moderate amount of overlap among the confidence regions of the group means of the CV scores (Figure 4c), but again the permutation tests produced results varying according to the test statistic (Goodall's *F* = 1.081, *p* = .339; Pillai's trace = 0.0877, *p* = .0080). The asymmetric component of shape variation provided a clearer differentiation among compass orientations, also mainly in the curvature of the basal contour of the blade (Figure 4b). There was only limited overlap among confidence ellipses of the CV scores (Figure 4d), and the permutation tests yielded highly significant results (Goodall's *F* = 2.615, *p* < .0001; Pillai's trace = 0.134, *p* < .0001).

By contrast, when relative orientation was considered, shape differences in the standards were less clear. Some differences in the symmetric component were associated with a more rounded or more pointed tip, and others again with the curvature of the basal part of the blade of the standard (Figure 4e). The confidence ellipses of the CV scores showed extensive overlap, with a slight tendency for the group means for the relative orientations of 60°, 180°, and 300° to be shifted to the left (in a 10 o'clock direction) by comparison to the means for 0°, 120°, and 240° (Figure 4g). The permutation test indicated no significant effects of relative orientation on the symmetric shape component of the standards (Goodall's *F* = 0.663, *p* = .894; Pillai's trace = 0.0499, *p* = .90). For the asymmetry component of shape in the standards, differences among the group means for relative orientations were subtle (Figure 4f). The confidence regions of CV scores for the groups overlapped substantially (Figure 4h), also with a slight tendency for the groups at 60°, 180° and 300° to be shifted away from the others (this time in a 4 o'clock direction in the plot). The permutation test found no significant effect of relative orientation on the asymmetric component of shape of the standards (Goodall's *F* = 1.287, *p* = .122; Pillai's trace = 0.0618, *p* = .478).

**Figure 5:**
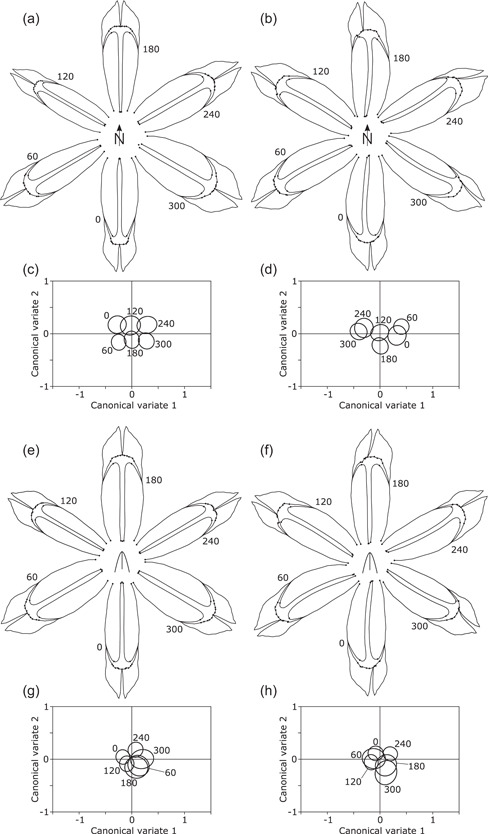
Effects of compass orientation (a–d) and relative orientation (e–h) on the shape of the style branches. The panels to the left (a, c, e, g) show results from analyses of the symmetric component of shape variation, whereas those to the right (b, d, f, h) display results from analyses of the asymmetric component. The diagrams of shape changes (a, b, e, f) show the differences among mean shapes for the different orientations exaggerated 15‐fold to make them more easily visible. Plots of canonical variate scores (c, d, g, h) show the 95% confidence ellipses for the means for the groups defined by compass or relative orientation.

The style branches varied considerably among compass orientations in the averages for the symmetric component shape, including widening or narrowing overall and also differentially between basal and apical regions (Figure 5a). The confidence ellipses for the average CV scores were adjacent to each other with only minor overlap (Figure 5b). In this plot, the compass orientations of 0°, 120°, and 240° appeared separated vertically above those of 60°, 180°, and 300°, and within both these groups, the positions were aligned in order from left to right. The permutation test again yielded differing results for the two test statistics (Goodall's *F* = 1.263, *p* = .211; Pillai's trace = 0.093, *p* = .0005). For the asymmetric shape component of the style branches, there was a clear systematic asymmetry resulting in an overall pinwheel symmetry, as well as clear differences between specific compass orientations (Figure 5b). The confidence ellipses for the means of CV scores were clearly separated but did not display an obvious pattern (Figure 5d), and the permutation test confirmed that the effects of compass orientation were highly significant (Goodall's *F* = 2.437, *p* < .0001; Pillai's trace = 0.117, *p* < .0001).

Relative orientation had a subtle effect on the symmetric component of the shape of the style branches, with those at 0°, 120°, and 240° appearing slightly narrower than those at 60°, 180°, and 300° (Figure 5e). This grouping was also visible in the scatter plot of CV scores, with the former group slightly offset from the latter in a 10 o'clock direction, but this pattern was obscured by the substantial overlap among the confidence ellipses for the mean CV scores (Figure 5g). The permutation test indicated no significant effect of relative orientation on the symmetric component of style branch shape (Goodall's *F* = 0.570, *p* = .904; Pillai's trace = 0.045, *p* = .935). The asymmetric component of shape variation showed a clear pinwheel symmetry, as well as some specific differences associated with some relative orientations (Figure 5f). The confidence regions for the mean CV scores overlapped extensively (Figure 5h), and the permutation test suggested no significant effect of relative orientation on the asymmetric component of shape of the style branches (Goodall's *F* = 0.972, *p* = .506; Pillai's trace = 0.0542, *p* = .645).

## 4 DISCUSSION

This study has replicated the main results of the preceding investigation (Tucić et al., 2018) and adds new insights due to the expanded study design. As in the earlier study, there was a systematic effect of compass orientation on the shape of floral organs and each individual floral organ is slightly and consistently asymmetric so that a rotational, or pinwheel symmetry resulted at the level of the whole flower. The new addition to the experimental design is that the present study also recorded the orientation of the outer spathe (Figure 1), which made it possible to compute the relative orientation of each floral organ. As it turned out, there was no clear effect of relative orientation on the floral organs. Here, we discuss these findings in the new conceptual context of the target phenotype (Nijhout & Davidowitz, 2003), which offers a useful general framework for thinking about concepts relating to asymmetry and phenotypic plasticity (Klingenberg, 2019), and the new idea of direction‐giving factors.

### 4.1 Directional asymmetry in response to anatomical position

This study found no clear effect of relative orientation on any of the three flower organs. This absence of consistent differences associated with relative orientation means that there is no evidence of directional asymmetry in relation to anatomical positions and therefore that anatomical position appears not to act as a direction‐giving factor for floral parts in *Iris pumila*. In other words, we found no significant deviation from the actinomorphic developmental bauplan expected for *Iris*, in which the floral organs in each whorl are all intrinsically equal.

This negative result raises the question of whether the anatomical position of floral organs relative to the spathes subtending each flower truly has no effect on shape, or whether an actual effect was missed because of some possible limitations in the experimental design and data. The compass orientations of spathes were distributed fairly equally among the six 60° sectors and frequencies did not significantly deviate from a uniform distribution. Accordingly, we can rule out a sampling bias preferring some overall orientations of flowers. By contrast, the relations between the orientations of the spathes and the floral organs were not uniform, with more than two‐thirds of flowers having a fall and style branch directly above the outer spathe, whereas somewhat less than one‐third were in an orientation rotated 60° from this, with a standard above the outer spathe. That one orientation prevailed is consistent with the expectation based on the ground plan of the *Iris* flower and information on its development (Figure 1b; e.g., Eichler, 1875; Pande & Singh, 1981; Remizowa et al., 2013). It is less clear, however, why there was variation in the orientation relative to the outer spathe. Possible reasons are that there might be genuine variation in the orientation of flower organs relative to the subtending spathes, that there might be a variable twisting of the long hypanthial tube in conjunction with the contort aestivation of the flowers, or imprecision in recording the orientations of outer spathes and floral organs. Regardless of why this variation exists, it raises the question of whether it may explain the lack of an effect of relative orientation on the shapes of floral organs. For all three floral organs, there was extensive overlap of the CV scores among the three relative orientations of the more frequent type of flower orientation, just as for those of the less frequent type (Figures 3g,h, 4g,h, and 5g,h). Likewise, the mean centroid sizes of all three floral organs were close to identical among the three relative orientations in the more common type as well as in the less common type (but there is a tendency for falls and standards to be slightly bigger in the less frequent type than in the more common type; right side of Table 1). Therefore, it seems even using just a single type of flower orientation in the analyses would not produce a stronger separation among relative orientations or change the conclusion that the relative orientation of floral organs has no discernible effect on their shape or size. Finally, the large sample sizes (Table 1) give us confidence that the absence of a significant effect of relative orientation is not due to a lack of statistical power; also, very subtle effects on the shape of floral organs have been demonstrated clearly and consistently for compass orientation in this study and its predecessor (Tucić et al., 2018).

A possible reason why shapes or sizes of floral organs did not differ according to anatomical orientation is that the flowers of *Iris pumila* grow in a terminal position and that therefore no adaxial–abaxial polarity may exist. The terminal position has also a special role in peloric flower morphs, where actinomorphic flowers appear in species that normally have zygomorphic flowers, thus involving a loss of differentiation among floral parts with different relative orientations, and which in many taxa only occurs in terminal flowers (Rudall & Bateman, 2003). *CYCLOIDEA*, one of the genes associated with the adaxial–abaxial polarity in flowers (Luo et al., 1996; Nakagawa et al., 2020; Spencer & Kim, 2018), has been found to be expressed in an asymmetric manner in meristems of terminal flowers in the *centroradialis* mutant of *Antirrhinum*, which induces peloric flowers in a terminal position, and therefore indicates that a prepattern for a floral polarity can exist even in a situation where it is not manifest in the mature flower (Clark & Coen, 2002). Similar information is not available for *Iris*. The available evidence is not sufficient to conclude whether the terminal flower position is the reason why relative orientation had no effect on the morphology of floral organs in *Iris pumila*, but this question might be answered by a similar study in a species with a branching inflorescence, which would provide a distinction between flowers in a lateral position, with clear adaxial–abaxial polarity, and flowers in a terminal position.

The finding that anatomical orientation has no detectable effect on the size or shape of floral parts in *Iris pumila* is consistent with expectations from its actinomorphic floral ground plan, but it also contrasts with the findings of other morphometric studies of asymmetry in flowers. Especially in relation to the adaxial–abaxial direction, clear directional asymmetry has been found by morphometric studies even in flowers that are superficially disymmetric (Savriama, 2018; Savriama et al., 2012). In zygomorphic flowers, of course, adaxial–abaxial differences are the dominant feature of floral architecture (Berger et al., 2017; Hsu et al., 2020, 2017; Soza et al., 2022; Wang et al., 2015) and are often so pronounced that morphometric studies treat repeated parts in more dorsal or ventral positions within a whorl as different structures altogether and focus exclusively on the symmetry and asymmetry in the left–right direction (e.g., Berger et al., 2017; Gardner et al., 2016; Sandner, 2020; Savriama, 2018). Therefore, the anatomical position is a direction‐giving factor that is important or even dominant for floral shape variation in many taxa and is inextricably related to floral architecture and development.

Anatomical position as a direction‐giving factor is not limited to flowers, but can also be recognized in different contexts, wherever repeated parts are arranged in a systematic way. The simplest case is bilaterally symmetric structures, where the left and right sides are repeated parts and developmental processes specifying left–right asymmetry are direction‐giving factors (Blum & Ott, 2018; Chitwood et al., 2012; Levin, 2005), but these considerations are more interesting for structures with more complex symmetry. Just as floral organs may differ consistently according to their positions in a whorl, different kinds of repeated parts may also vary systematically in their target phenotypes according to the position. Examples of this type are systematic changes associated with the sequence of leaves along with a shoot (heteroblasty; e.g., Chitwood et al., 2016; Jones, 1993), where the target phenotype of leaves varies predictably in response to their position along with a shoot or according to the time of initiation of the respective primordia. Likewise, similar patterns also occur for variation among flowers within inflorescences (Bateman & Ruddall, 2006). Although these cases usually are not considered to be instances of directional asymmetry, the iteration of parts such as leaves on a shoot (or an entire plant) or flowers in an inflorescence can be interpreted as translational symmetry (Savriama & Klingenberg, 2011), and therefore systematic morphological differences between them are a type of directional asymmetry (the same reasoning applies to serial homology of structures repeated along the anterior–posterior axis of animals; Savriama et al., 2017). The relative positioning of primordia in meristems, along with its consequences on the distribution of active substances such as auxin, can also have systematic effects on the left–right asymmetry of individual leaves (Chitwood et al., 2012; Martinez et al., 2016), or the *CYC2*‐like alleles can affect shapes and asymmetries of floral parts (Hsu et al., 2017). Altogether, both for flowers and for other structures, there are many possible ways in which iterated parts developing in different anatomical positions can experience different conditions and therefore have distinct target phenotypes so that the resulting systematic differences constitute directional asymmetry. Some of these types of directional asymmetry may be found by the morphometric approaches routinely used to study asymmetry, but many require specific study designs to be investigated empirically.

Anatomical position as a direction‐giving factor also relates to the concepts of positional information and positional signaling (Jaeger & Reinitz, 2006; Wolpert, 1969; Xu et al., 2021). By providing cells and tissues of developing organs with the specific identities according to their anatomical positions, patterning processes act as direction‐giving factors specifying differences among target phenotypes in the resulting mature structures, which are observable as directional asymmetry. In the best‐understood examples, direction‐giving factors can therefore be equated to known developmental mechanisms, as is the case for adaxial–abaxial asymmetry in flowers (Nakagawa et al., 2020; Spencer & Kim, 2018) and for left–right asymmetry in animals (Blum & Ott, 2018; Grimes & Burdine, 2017). Yet, because serially repeated structures are instances of translational symmetry, there is no fundamental difference between developmental processes that establish symmetry or asymmetry and those that form other aspects of the bauplan of structures such as flowers or entire organisms. Therefore, direction‐giving factors for asymmetries associated with different anatomical positions may not differ fundamentally from developmental signals or patterning processes in other contexts.

### 4.2 Directional asymmetry in response to consistent external gradients

This study found that the shapes of floral organs differed among compass orientations, replicating the results of the preceding investigation (Tucić et al., 2018). For all three flower organs, the systematic differences due to compass orientation were clearer for the asymmetric than the symmetric component of shape, also in agreement with the previous findings. Nevertheless, plots of the group averages of the first two CV scores for the different compass orientations (Figures 3c,d, 4c,d, and, 5c,d) did not display patterns that related directly to those orientations, like those in the previous study did (Tucić et al., 2018, figs. 4c,d, 5c,d, and 6c,d). All these effects were subtle, and therefore the differences in results might originate from various minor changes, for instance, that the present study (with 462 flowers from 314 plants) was not as near‐perfectly balanced as the previous experiment (two flowers were collected from each of 267 plants, oriented so that one had a fall and the other a standard in a southerly direction). The key addition in the present study is the direct information on the anatomical position of the flowers through recording the compass orientation of the outer spathes. These compass orientations were spread fairly evenly over all six 60°‐sectors in a way that was not distinguishable from a uniform distribution. This confirmed that not only the pots in which the plants were grown were positioned haphazardly, without any reference to compass orientation, but also that the anatomical orientations of the flowers themselves were effectively randomized. This confirmation further strengthens the inference that the observed effect of compass orientation on the shapes of floral organs must be due to phenotypic plasticity in response to an extrinsic gradient affecting all flowers in a consistent manner. As argued before (Tucić et al., 2018), the factor that is by far the most likely to be responsible for this effect is solar irradiance.

This reasoning indicates that environmental gradients affecting plants in a consistent manner can be direction‐giving factors responsible for directional asymmetry. The mechanism by which such directional asymmetry originates is a reaction norm that translates the environmental gradient into observable phenotypic variation. Because a reaction norm is a change in the target phenotype in response to a change in an environmental parameter (Klingenberg, 2019; Nijhout & Davidowitz, 2003), the response to a gradient in the environmental parameter that consistently affects every plant is a systematic change in the target phenotype among floral parts according to their orientations and shared by all the plants. This means that such variation meets the criteria for directional asymmetry. Note, however, that this requires a homogeneous environmental gradient and a consistent orientation of all flowers in relation to the gradient. If the gradient contains heterogeneities, it will generate a component of fluctuating asymmetry in addition to the shared component of directional asymmetry. Also note that, because most studies do not record compass orientation of flower parts (or orientation in relation to possible other environmental gradients), this type of asymmetry would usually be included as a component of fluctuating asymmetry (random differences among parts within each flower; for more detailed discussion, see Tucić et al., 2018).

### 4.3 Directional asymmetry of individual parts

A further result is the consistent pattern of “pinwheel symmetry” that was evident for the averages of the asymmetry component of shape in all three floral organs (Figures 3b,f, 4b,f, and 5b,f). This is a systematic asymmetry of individual flower organs that affects all orientations similarly, thereby producing the “pinwheel” pattern in the diagrams. This pattern was less clear for the style branches than for the falls and the standards, possibly because no landmarks were located in the most distal portion of the style branches, and changes, therefore, are entirely extrapolated by the thin‐plate spline from the arc of landmarks at the stigma to the more distal parts (Figure 5b,f). The pattern of pinwheel symmetry also confirms a result from the preceding study (Tucić et al., 2018), and systematic directional asymmetry of each individual organ was also identified in other analyses (Radović et al., 2017).

It is plausible that this pattern relates to the contort aestivation, the way in which floral organs are rolled up in the bud in a consistent direction (Endress, 1999). In *Iris*, the falls are consistently rolled up in a right‐hand direction (dextrorse, counterclockwise when viewed from above; Figure 1b; Eichler, 1875; Guo & Wilson, 2018; Schoute, 1935). This means that systematic differences exist between the left and right halves: for each fall the left margin is situated inside and the right margin outside the neighboring falls in the bud (Figure 1b), which requires an inherent morphogenetic difference between the left and right sides of each fall. Also, as a consequence, the right margin of each fall is exposed to the outside, whereas the left margin is not, which may have observable morphological effects (Endress, 2008). Whether as a result of an inherent asymmetry between the left and right sides of each fall or due to the difference in exposure, the observed asymmetries in the shapes of individual falls (Figure 3b,f) are likely to be related to their contort aestivation. Therefore, this type of aestivation, or more generally the arrangement of repeated organs as part of floral architecture, can act as a direction‐giving factor and produce a distinct level of directional asymmetry within those organs.

How the standards and style branches are arranged in flower buds differs among *Iris* species (Eichler, 1875; Guo & Wilson, 2018). For *Iris pumila*, the cross‐sections through flower buds that we have examined show that the standards do not overlap but are confined laterally between the “beards” in the midlines of the adjacent falls, as for other species with midline elaborations of the falls (Guo & Wilson, 2018), whereas the style branches are positioned “back to back” in the center of the bud (Figure 1b). Both the median regions of the standards and the margins of the style lobes appear to be tightly packed together and against the inner (left) halves of the falls. Lateral expansion of the falls may therefore produce rotational forces on the adjacent structures inside the bud. The median ridges and the markedly involute margins of the style branches tend to be positioned inside the buds in a manner suggestive that some twisting may occur, but this seems to be fairly weak and the arrangement is not entirely regular. The origin of the asymmetries of individual standards and style branches is, therefore, less clear than it is for the falls, but it might also be related to the asymmetry of the falls. Furthermore, the bases of the falls and standards are united into a perianth tube, providing an opportunity for developmental interactions. Morphogenetic effects in response to mechanical forces have been widely found and mechanisms identified for them (Braam, 2005; Bull‐Hereñu et al., 2022; Hervieux et al., 2016; Trinh et al., 2021). In the present context, asymmetries imparted in this way on the standards and style branches could be considered as a subtle form of what Endress (2008) called “imprinted shape.” Note, however, that any such interpretation must remain tentative until more direct evidence becomes available.

### 4.4 Direction‐giving factors and the nature of directional asymmetry

This study has distinguished three types of directional asymmetry corresponding to different direction‐giving factors: anatomical position at the whole‐flower level, a consistent environmental gradient, and asymmetries of individual organs due to their arrangement relative to one another. Separating the direction‐giving factors has been possible in *Iris pumila* because multiple types of directional asymmetry exist due to the complex symmetry of the flowers (Klingenberg, 2015; Savriama & Klingenberg, 2011), and recording the relative orientation and compass orientation of floral parts as well as the bilateral symmetry of individual organs enabled us to separate the effects of multiple direction‐giving factors (Figure 1). For structures with bilateral symmetry, those direction‐giving factors also may apply, but their effects all contribute to the same left–right asymmetry and are therefore much more difficult to disentangle from each other.

The direction‐giving factors differ from each other fundamentally in how they produce phenotypic differences among floral organs and therefore directional asymmetry. The whole‐flower asymmetry due to anatomical position, which was not detectable in this study but is prominent in the adaxial–abaxial asymmetry of flowers in many other plants (Berger et al., 2017; Savriama et al., 2012; Wang et al., 2015), is clearly an intrinsic effect based on known mechanisms of positional specification (Nakagawa et al., 2020; Smyth, 2018; Spencer & Kim, 2018). Likewise, the pinwheel pattern of consistent directional asymmetries within each floral organ is an intrinsic effect, most likely related to the growth of floral organs and their arrangement and interactions in the developing flower buds. By contrast, the directional asymmetry associated with compass orientation clearly must be due to an environmental gradient (most likely solar irradiance; Tucić et al., 2018) and is therefore of extrinsic origin. Accordingly, differences among the target phenotypes of parts according to position within a flower can arise via fundamentally different mechanisms, whose effects can be combined and superimposed one onto another.

The inference that directional asymmetry can arise in different ways, both intrinsic and extrinsic to the organism and structure under study, may be surprising. Especially, this may seem at odds with the often‐repeated view that directional asymmetry has a genetic basis (e.g., Leamy, 1984; Palmer & Strobeck, 1992; Van Dongen, 2006; Van Valen, 1962). It is helpful to recall that all parts of a flower share the same genome and that the observed differences among parts are therefore due to the capacity of the genome to produce different phenotypic outcomes in the precursors of those parts that experience different developmental contexts. Directional asymmetry has a genetic basis because the genome influences how the target phenotype differs according to the different developmental contexts of repeated parts in different positions or how it responds to external conditions through reaction norms. Yet, even though developmental processes clearly are under the control of the genome that encodes resources such as various proteins and RNAs directly involved in the process, positional information or extrinsic inputs from the environment can modulate these processes. If such modulation leads to systematic differences in the phenotypic outcomes of repeated parts according to their positions, this means that those parts differ in their target phenotypes and we observe directional asymmetry. Because this origin of directional asymmetry is similar to the developmental bases of other forms of developmental plasticity, there is a close link between the responses of the target phenotype to positional changes in adaxial–abaxial or left–right directions for directional asymmetry and the responses to proximal–distal position for heteroblasty or the differentiation according to anterior–posterior position in serial homology (Klingenberg, 2019). Considering directional asymmetry in close connection to those related concepts is helpful for understanding its development and evolution.

This study is unusual because the orientations of all flowers were recorded. This is done very rarely for compass orientation (Tucić et al., 2018), but it is not always feasible even for the anatomical position, for instance in actinomorphic flowers when no clear markers of adaxial–abaxial orientations are available (e.g., Frey et al., 2007; Neustupa, 2020). In such a situation, it is impossible to separate directional and fluctuating asymmetry and analyses, therefore, need to consider a single component of total asymmetry (Klingenberg, 2015). This overall measure of asymmetry, sometimes called “radial asymmetry,” is usually treated like fluctuating asymmetry and used as a measure of developmental instability (e.g., Frey & Bukoski, 2014; Neustupa, 2020; Perfectti & Camacho, 1999; Siikamäki & Lammi, 1998; Tucić et al., 2008). This is not a serious problem in practice if directional asymmetry is only a minor proportion of total asymmetry, but of course, it complicates the interpretation of the results (Palmer & Strobeck, 2003; Tucić et al., 2018). It is unsettling, however, that it is unclear whether the direction‐giving factors covered in this study, more than in any previous investigation, really include all the possible factors. Also, in most other study systems, the same problem is likely to apply but there is no possibility of teasing apart the effects of different direction‐giving factors; instead, those effects will appear partly as directional and partly as fluctuating asymmetry, in proportions that cannot be fully understood.

## CONFLICTS OF INTEREST

The authors declare no conflicts of interest.

## Data Availability

The raw data used in this paper are archived in the Zenodo repository, DOI:10.5281/zenodo.5546306.
